# A Perspective on the Role of Point-of-Care “Immuno-Triaging” to Optimize COVID-19 Vaccination Distribution in a Time of Scarcity

**DOI:** 10.3389/fpubh.2021.638316

**Published:** 2021-08-03

**Authors:** Yi Zhang, Angela Rogers, Kari Nadeau, Jun Gu, Samuel Yang

**Affiliations:** ^1^Sino-Singapore International Joint Research Institute, Guangzhou, China; ^2^School of Mechanical and Aerospace Engineering, Nanyang Technological University, Singapore, Singapore; ^3^School of Medicine, Stanford University, Stanford, CA, United States; ^4^Department of Cardiovascular Surgery, West China Hospital, Sichuan University, Chengdu, China

**Keywords:** COVID-19, vaccine, point-of-care, immunodiagnostics, vaccine distribution

## Abstract

Vaccine bears hope to bring COVID-19 pandemic under control. With limited supply, vaccines must be utilized efficiently to provide protection to those who need it most. Currently, no practical framework has been proposed to ensure fair vaccine allocation at individual level, which is a recognized problem. We propose here an evidence-based decision-making framework for COVID-19 vaccine appropriation that prioritizes vaccine doses to individuals based on their immunological status, or immuno-triaging. To ensure successful implementation of the proposed framework, point-of-care (POC) immunodiagnostic testing is needed to quickly ramp up the testing capability. Considerations for deploying POC immunodiagnostic testing at such a large scale are discussed. We hope that the proposed immunological decision-making framework for evidence-based COVID-19 vaccine appropriation provides an objective approach to ensure fair and efficient utilization of the scarce vaccine resource at the individual level that also maximizes the collective societal benefit.

## Introduction

Since its onset in January, 2020, COVID-19 has caused millions of deaths worldwide (1, 2). Several new SARS-Cov-2 variants have caused the death toll to increase rapidly in recent waves (3). In the current dire situation, COVID-19 vaccine finally brings a glimmer of light at the end of the tunnel. As of Jun 4^th^, 2021, a total of 102 COVID-19 vaccine candidates are under clinical evaluation (4), and 9 Emergency Use Listing (EUL) are issued by the World Health Organization (WHO) (5). Armed with these vaccines, the world will be ready to enter the second half of the battle against COVID-19 pandemic.

Without effective pharmacological interventions, molecular testing (nucleic acid amplification test) has been our best defense against COVID-19 to date (6). Besides molecular testing, a wide range of immunodiagnostic tests have been developed to detect the IgG and/or IgM against SARS-CoV-2, the viral pathogen that causes COVID-19. Emerging evidences suggest that T-cell immunity may play an equal, if not greater, role in protective immunity against SARS-CoV-2 (7, 8). As public health strategies shift toward vaccine and immunity, we believe POC immunodiagnostic should play a major role in vaccine appropriation in the second half of the battle against COVID-19.

Despite the unprecedented speed and scale of R&D effort in COVID-19 vaccine development, the supply of vaccines will be limited. Even though an ambitious goal of 1 billion doses by the end of 2020 is planned globally (9), this number is far from sufficient. It will take at least another year to produce enough doses for the world. In reality, some of the vaccine candidates in the production plan may not cross the finishing line, and some of the planned manufacturing capacity may be delayed due to disruption of supply chains. Even under optimal circumstances, the massive demand will put tremendous pressure on the global supply of biomedical products needed for vaccine production. Concerns have already been raised on potential shortage of horseshoe crab's blood (10), glass vial (11), and syringe (12) that are required for vaccine testing, storage and administration. In short, COVID-19 vaccine will be a scarce resource.

With limited supply for the first few months and likely years, a critical question is who gets the vaccine first. This is not an easy question to answer. To bring the COVID-19 pandemic under control in the shortest possible time, vaccines must be utilized efficiently to provide protection to those who need it most. Most frameworks proposed to guide equitable allocation of vaccines are primarily focused on targeting population groups (13, 14). For example, the WHO's fair allocation framework through COVAX is focused on mortality reduction and protection of health system by targeting groups including frontline healthcare works and age >65 with high risk factors (15). But only a limited number of practical frameworks, such as the allocation plans employed by individual US CDC jurisdictions (16), have been proposed to ensure more precise vaccine allocation at the individual level, which is a recognized problem (13). Moreover, because the development of multiple vaccine candidates is occurring in isolation and in parallel to compress the usual vaccine timeline from 10-15 years to 1–2 years, crucial information regarding the efficacy, longevity, safety, and deployment of the various vaccines will be variable, asynchronous, and evolve over time (17). Those who received early generation of vaccine with rapidly waning immune responses may require re-vaccination using an improved second-generation vaccine. There are patient-level differences in susceptibility to SARS-CoV-2 infection, variable immunity in asymptomatic individuals or those recovered from COVID-19, and emerging evidence of pre-existing immunity resulting from past exposure to other human coronaviruses (18).

Although there is no consensus on the correlation between seropositivity and protective immunity against SARS-CoV-2, immunodiagnostic testing is still the primary metric used to evaluate the efficacy of COVID-19 vaccines, and the decision on the necessity of booster vaccine is also based on the antibody titer (19, 20). As such, we propose here an immuno-triaging framework for both fair and precise COVID-19 vaccine appropriation in a time of scarcity that incorporates existing frameworks that target priority populations but also accounts for the immunological status of each individual (20). Due to the large testing scale and relatively simple assay format, POC immunodiagnostic testing, particularly in community settings and primary healthcare settings, can play a central role in establishing an equitable and evidence-based vaccine distribution at the individual level. The necessity and considerations for large-scale deployment of POC immunodiagnostic testing for immuno-triaging are discussed in this perspective. We hope that it could assist in making objective decisions based on scientific and medical evidence and lead to the most equitable and efficient utilization of a limited vaccine supply for the collective benefit of society.

## Immuno-Triaging for Evidence-Based COVID-19 Vaccine Appropriation

**Figure 1:**
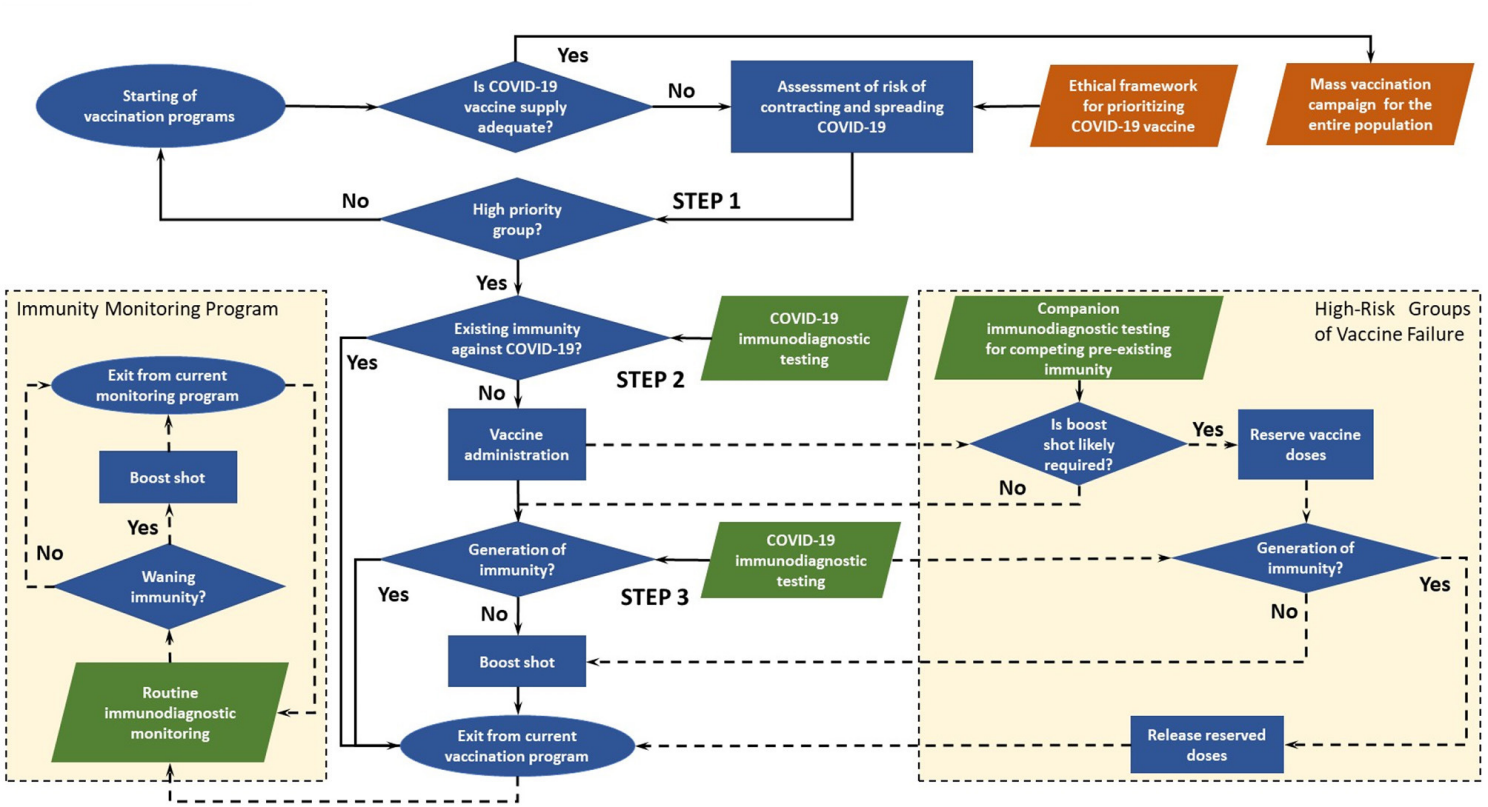
Decision-making flowchart for immuno-triaging of COVID-19 vaccine.

The decision-making flowchart of immuno-triaging developed by us is illustrated in Figure 1. When in ample supply, vaccines are administered without testing pre-existing immunity in mass vaccination campaigns. In the case of COVID-19 vaccine, which has an urgent and huge worldwide demand but limited supply in the foreseeable future. The collective benefit of immuno-triaging would outweigh the cost of immunodiagnostic testing when such testing is accessible. In this framework proposed by us, POC immunodiagnostic testing ensures equitable vaccine allocation, both on an individual level and societal basis. However, getting access to immunodiagnostic tests may be no less challenging than getting access to vaccines, which must be taken into consideration when implementing the framework. Here, Immunodiagnostic testing refers to serological tests or T-cell immune response tests.

There are three key steps in the main immuno-triaging decision path, and two side decision paths for high-risk groups of vaccine failure and immunity monitoring program post vaccination.

### Main Decision Path

***Step 1: Assess risk of contracting and spreading COVID-19 and identify*** ***high-risk groups to enroll in an active vaccination program***
  Prioritized access to vaccines should be given to groups with high risks of contracting and spreading the disease. The risks may be assessed by individual's baseline medical conditions and social risk factors using metrics such as social vulnerability index (SVI) (21). Low-risk groups in the initial assessment will be enrolled in future program when vaccines become more readily available. The assessment could be integrated into existing ethical frameworks (13–15) for the collective benefit of people by ensuring that certain groups are not disadvantaged due to morally irrelevant factors such as religion and race. Low-priority group will be re-admitted to the vaccination program when more vaccine doses are available.
***Step 2: Assess existing immunity against COVID-19 in prioritized groups*** ***immuno-triaging of COVID-19 vaccines***
  Vaccines are appropriated according to immunodiagnostic evidence based on virus-specific antibodies, neutralizing antibodies, or in ideal scenario T-cell immune response if condition allows. Qualitative testing based on ELISA or competitive ELISA could be used to determine the presence of IgM/IgG pr against SARS-CoV-2 (8, 18, 22–24). The cutoff level for protective immunity could be determined based on models like the one suggested by Khoury et al. (25). Vaccines are prioritized for individuals without existing immunity against COVID-19. Individuals with existing immunity due to virus exposure are relieved from current vaccination program. This group would be included in the immunity monitoring program, one of the side decision paths.
***Step 3: Assess immune response and determine whether a booster vaccine is*** ***required***
  The efficacy of vaccines in clinical trials is evaluated by antibody titer or T-cell immune response, and the same strategy could be used to assess immune response in the framework. Although multiple doses are suggested for a number of vaccine candidates, it has been shown in clinical trials that the antibody titer in certain individuals reaches the threshold for seroconversion with only a single dose (19). These individuals could skip subsequent doses when vaccine supply is limited, and catch-up doses may be given if necessary when immunity wanes or vaccines become widely available. On the flip side, clinical trials for single-dose vaccine candidates reveal that booster vaccine may be required for individuals with pre-existing immunity against vaccine vectors (20). Therefore, to ensure efficient utilization of vaccines, the booster should be appropriated based on immunodiagnostic evidence.

### High-Risk Groups of Vaccine Failure: Assess Pre-existing Immunity That Predicts the Likelihood of An Individual Requiring a Booster Vaccine

POC immunodiagnostic testing could be used to identify individuals who are predicted to require a booster vaccine based on immunodiagnostic evidence (20). In such a scenario, a dose is reserved to ensure the booster vaccine is available so that the first dose does not go waste. Immunodiagnostic testing should be conducted to check the immune response before the administration of the booster vaccine. If the individual successfully generates immunity without the booster vaccine, the reserved vaccine dose is released. Individuals who receive the booster vaccine could be included in the immunity monitoring program.

### Immunity Monitoring Program: Tracking Immune Response and Checking for Waning Immunity

Routine immunodiagnostic monitoring tracks the dynamics of immune response post vaccination and identifies individuals who may no longer have protective levels of antibody or T-cell memory. Combined with cloud-based information platform, big-data tools (26), and other digital solutions (27, 28), routine measurement of these levels could guide future vaccination programs and public health responses. If immunodiagnostic testing results impart waning immunity, booster vaccine could be administered to these individuals. After receiving the booster vaccine, Individuals may exit or remain in the routine monitoring program depending on available resources.

## Essential Role of POC Immunodiagnostic Testing in Implementing the Framework

POC immunodiagnostic testing is imperative for successful implementation of the framework. Currently, immunodiagnostic testing, particularly POC immunodiagnostic testing, is only recommended as a surveillance tool or a primary screening mechanism to supplement molecular testing (29). Molecular testing will continues serving as the primary diagnostic tool for patients acutely ill or exposed to COVID-19, but immunodiagnostic testing will be a key tool in immuno-triaging of COVID-19 vaccines.

Widespread deployment of POC immunodiagnostic testing would be prioritized because of the following considerations.

### POC Immunodiagnostic Testing Will Enable Efficient COVID-19 Vaccine Utilization

To bring COVID-19 under control calls for efficient utilization of the scarce vaccine resource. Rapid test-to-decision workflow is of the outmost importance in the context of immuno-triaging. POC testing brings the testing capability to the site of patient care and offers sample-to-answer tests that require minimal user intervention. Besides reduced cost and demand for resources compared to centralized testing, one defining characteristic of POC testing is the rapid access to testing results for timely decisions on COVID-19 vaccine appropriation. Short wait time also eases patients' anxiety level, reduces the number of clinical visits (which is critical for vaccine adherence), and decreases the chance of infection while waiting for testing results.

### Immunodiagnostic Testing Needs to Be Conducted at a Large Scale That Is Beyond the Capability of Existing Centralized Testing

As the focus of testing for COVID-19 shifts from identifying pathogen to determining immunity in the second half of the battle, immunodiagnostic testing will need to be conducted at a large scale over wide geographic regions. The huge test volume and scattered population distribution present a logistical nightmare for centralized testing schemes: sample delay, loss and mislabeling are inevitable when tests are conducted on such a large scale. If communication of testing results requires a long turnaround time, decisions on vaccine appropriation will be delayed. Decentralization will enable rapid ramp-up in immunodiagnostic testing capability and relieve the burden on central healthcare facilities. In regions with poor medical resource, POC immunodiagnostic testing may be the only viable option for implementing the proposed framework for fair and efficient vaccine utilization.

### Large-Scale POC Immunodiagnostic Testing Is Feasible and Relatively Easy to Implement

The proposed immuno-triaging framework requires immunodiagnostic evidence at various stages over a period of several weeks or even months. Therefore, a high level of patient's compliance with the testing schedule is essential for efficient vaccine utilization. POC immunodiagnostic testing could enhance patient's adherence to the program due to its easy access, rapid turnaround time and timely clinical decision. In fact, COVID-19 testing based on POC immunoassays has already been deployed in large scale in Singapore (30). The decentralized arrangement makes active follow-up with patients a relatively easy task. Local recruits could establish effective partnership with local communities to promote the COVID-19 vaccination program for improved outcomes.

### POC Immunodiagnostic Testing Enhances Patient and Community Engagement in COVID-19 Vaccination Program

The proposed immuno-triaging framework requires immunodiagnostic evidence at various stages over a period of several weeks or even months. Therefore, a high level of patient's compliance with the testing schedule is essential for efficient vaccine utilization. POC immunodiagnostic testing could enhance patient's adherence to the program due to its easy access, rapid turnaround time and timely clinical decision. The decentralized arrangement makes active follow-up with patients a relatively easy task. Local recruits could establish effective partnership with local communities to promote the COVID-19 vaccination program for improved outcomes.

## Concerns With Existing COVID-19 POC Immunodiagnostic Tests and Gaps to Close

### Test Accuracy

**Figure 2:**
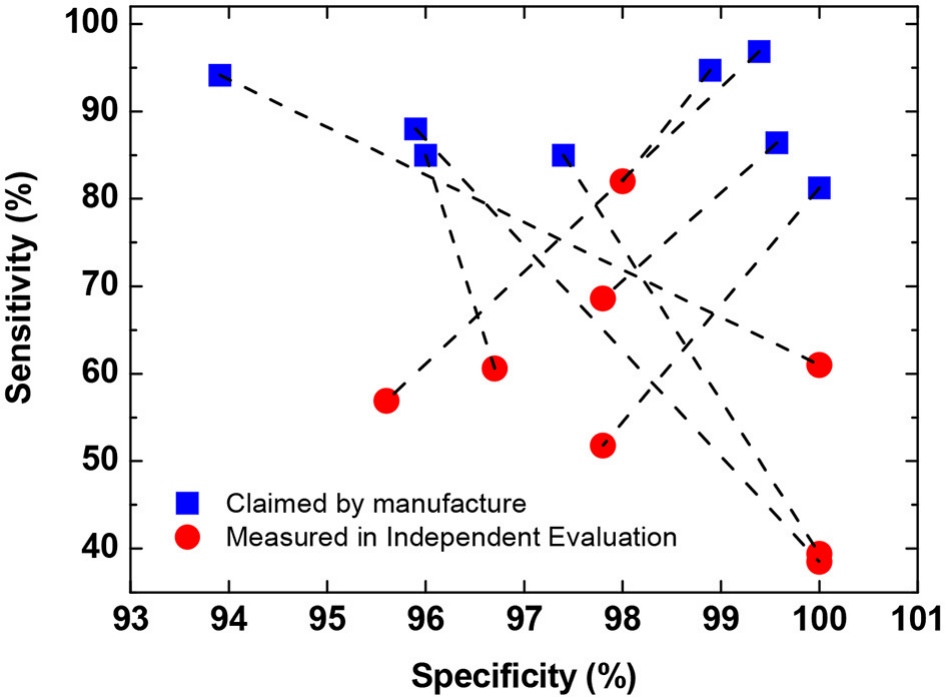
Comparison of self-claimed and independently evaluated performance of eight POC immunodiagnostic tests. Data obtained from (31). Data in the plot show the mean sensitivity and specificity for total antibody detection (either IgM or IgG). In cases where the performance for IgM and IgG are reported separately by the manufacture, the lower values are used in the plot.

One major concern about widespread deployment of POC immunodiagnostic testing is the accuracy of test kits. At the moment, the majority of POC immunodiagnostic testing kits are based on lateral flow assay (LFA), and many of them show suboptimal performance in independent evaluations. Australian Therapeutic Goods Administration (TGA) has announced that all eight POC immunodiagnostic test kits evaluated in its Post Market Review so far “have claimed a better sensitivity than that observed” in its independent evaluation (Figure 2) (31). It is worth noting that majority (>50%) of the samples in the cohort used for sensitivity evaluation by TGA are from early-stage infections ( ≤ 14 days) which are expected to lead to lower sensitivity. Nevertheless, even when tested with all late-stage samples (> 14 days), only two out of the eight tests show a similar performance to that claimed by the manufacturers.

A meta-analysis of COVID-19 immunodiagnostic testing reveals that the sensitivity of LFA is significantly lower than that of ELISA and chemiluminescent immunoassays (CLIA) (32). Both ELISA and CLIA could provide quantitative immunodiagnostic measurements. Although CLIA has a higher sensitivity than ELISA, it also shows a significantly larger variability. Compared to CLIA, ELISA is less resource demanding and easier to translate into POC testing using simple microfluidic systems such as magnetic digital microfluidics. POC ELISA could improve the accuracy POC immunodiagnostic testing and offer quantitative antibody titer measurements to monitor immune response post-vaccination.

Existing immunodiagnostic tests are optimized for a high positive predictive value, which means they are designed to ensure the positive results are true positive. And most of these tests have a low negative predictive value, which means there could be a relatively large number of false negatives (31, 33). The framework appropriates vaccine doses based on negative immunodiagnostic results. Hence, POC immunodiagnostic tests with high negative predictive values should be selected for pre-vaccination screening. An orthogonal testing algorithm could be implemented in regions with a high COVID-19 prevalence where the tests are likely to have a low negative predictive value to ensure the negative results are true negative.

### Lack of POC Testing for COVID-19 T-Cell Immune Response

Recent studies reveal that CD4 and CD8 T-cells respond to multiple SARS-CoV-2 proteins and “memorize” the immunity for a longer duration than antibodies (7, 8), suggesting that T-cell response could potentially serve as a more accurate biomarker for COVID-19 immunity than antibody titer. Effort has already been put into developing lab-based centralized T-cell testing for COVID-19 immunity check, and one has obtained emergency use authorization from US Food and Drug Administration (FDA) (34). Commonly used T-cell detection assays include flowcytometry and enzyme-linked immune absorbent spot (ELISpot). However, these assays are not readily translatable for POC testing. Sample preparation presents the greatest challenge for POC T-cell immunity testing. In the case of ELISpot, sample preparation could be accomplished by separating CD4 and CD8 T-cells from whole blood using immuno-conjugated magnetic particles. Assays that rely on magnetic particles can be readily translated to POC testing by using magnetic digital microfluidics. While these proposed approaches of POC T-cell immunity testing are feasible, it still presents a great challenge due to the complex assay format, and resource must be devoted to validate and optimize these testing for clinical use.

### Considerations for Implementing POC Immunodiagnostic Testing

POC is an umbrella term that describes a wide variety of healthcare settings. Applicable scenarios for POC immunodiagnostic testing should be defined by answering “where is the point,” “who to care,” and “what and how to test” (35). In the context of POC immunodiagnostic testing for COVID-19 vaccine appropriation, we have categorized POC settings in three classes and summarized them in Table 1 according to resource availability and testing requirements. While the framework already dictates “who to care” and “what to test,” we need to examine “where” and “how” to conduct POC immunodiagnostic testing.

**Table 1:** Settings and applicable scenarios for POC immunodiagnostic testing.

	**Category I**	**Category II**	**Category III**
POC settings (where is the point)	Self-testing in home care setting	IIa: Community with adequate resource IIb: Community with poor resource	IIIa: Primary healthcare setting IIIb: Bedside, examination room, emergency department in full-fledged hospital
Considerations for testing implementation (how to test)	- Certain level of expertise is required to conduct POC immunodiagnostic testing - Certain risks associated with handling potential contagious biosamples	- Relatively intensive training is required due to the general lack of medical background in local recruits - Select suitable POC immunodiagnostic testing according to local resource availability (e.g., electricity, cold chain, etc.) - Conduct Inspection at high frequency - Develop protocol for biohazard disposal according to local environment and resource	- Conduct necessary training - Integrate POC immunodiagnostic testing into existing medical managing systems
Recommendation	POC immunodiagnostic testing is not recommended for self-testing except for self-sample-collection	LFA is recommended for POC immunodiagnostic testing in Category IIa and IIb. Quantitative POC immunodiagnostic testing is recommended for IIa. POC testing for T-cell immune response is recommended for Category IIa if conditions permit.	Use POC immunodiagnostic testing as a supplement. Centralized testing should be given the priority if it is easily accessible. POC testing for T-cell immune response is recommended for Category IIa if conditions permit.

POC immunodiagnostic testing is recommended for community (Category II) and limited healthcare (Category III) settings. However, centralized testing should be given the priority if it is easily accessible in Category III settings. POC immunodiagnostic testing is not recommended for self-testing in home care settings (Category I) because a certain level of expertise is required to handle the sample, conduct the test and interpret the results. However, users may collect the testing on their own and send the samples for testing in community testing center or primary healthcare facilities.

To ensure the reliability of POC testing, training must be provided to local recruits, and routine inspection should be conducted to ensure test procedures are standardized, devices are calibrated and test kits are properly stored. Other considerations for POC immunodiagnostic testing include proper biohazard waste disposable protocol. Standard biohazard disposable protocol is usually already installed in Category III settings. Local sources and environment should be taken into consideration when establishing biohazard disposable protocol in Category II setting.

## Conclusion and Outlook

The rapid pace of COVID-19 vaccine development brings hope to bring the pandemic under control. But the huge discrepancy between supply and demand means that difficult decision must be made on how to allocate this scarce resource in the way that is both fair and most efficacious (16). In this perspective, we propose an immuno-triaging framework for evidence-based COVID-19 vaccine appropriation. The implementation of the framework and the role of POC immuno-testing in the framework are described in detail, and the concerns with existing COVID-19 POC testing are also discussed. We hope the proposed framework could provide an objective approach to ensure fair and efficient utilization of the scarce vaccine resource at the individual level that also maximizes the collective societal benefit.

Accurate and precise POC immunodiagnostic testing could be a key tool in vaccine immuno-triage. Nonetheless, many existing POC immunodiagnostic tests only measure antibody response and are plagued by poor performance. Better POC tests and testing algorithms are needed to implement the proposed framework. New POC immunodiagnostic testing for T-cell immune response could further improve the identification of patients who do (and don't) need further booster vaccines. A cloud-based centralized information system to coordinate decentralized vaccination centers will provide the digital infrastructure to ensure the successful implementation of the proposed framework. We encourage both industry and academia to prioritize the development of POC immunodiagnostic tests for the second half of the battle against COVID-19.

## Data Availability Statement

The original contributions presented in the study are included in the article/supplementary material, further inquiries can be directed to the corresponding author/s.

## Author Contributions

YZ and SY conceptualized the framework and wrote the manuscript. AR, KN, and JG improved the framework and edited the manuscript. All authors contributed to the article and approved the submitted version.

## Conflict of Interest

YZ declares equity interest in DropLab Scientific Ltd. DropLab Scientific is a startup company founded in Guangzhou, China in 2020. It is a spinoff of Sino-Singapore Joint International Research Institute and Nanyang Technology University Singapore. DropLab Scientific aims to commercialize magnetic droplet microfluidic technology for a wide range of applications such as energy harvest, in vitro diagnostics, and manufacturing. The remaining authors declare that the research was conducted in the absence of any commercial or financial relationships that could be construed as a potential conflict of interest.

## Publisher's Note

All claims expressed in this article are solely those of the authors and do not necessarily represent those of their affiliated organizations, or those of the publisher, the editors and the reviewers. Any product that may be evaluated in this article, or claim that may be made by its manufacturer, is not guaranteed or endorsed by the publisher.
